# Editorial: Structure-function metrology of proteins

**DOI:** 10.3389/fmolb.2023.1125791

**Published:** 2023-01-17

**Authors:** Alex R. Jones, Isabel Moraes, Milena Quaglia

**Affiliations:** ^1^ Biometrology, Chemical and Biological Sciences Department, National Physical Laboratory, Teddington, United Kingdom; ^2^ NML at LGC Ltd., Teddington, United Kingdom

**Keywords:** protein metrology, biologics, higher order structure, protein function, protein engineering

The importance of a protein’s structure to its function is beyond doubt. The Protein Databank (PDB) (Berman et al., 2003) and the AlphaFold Protein Structure Database (https://aplhafold.ebi.ac.uk), based on the AlphaFold algorithm (Jumper et al., 2021), make freely available vast numbers of experimentally-determined and predicted 3D protein structures that underpin much impactful science. What is becoming increasingly clear, however, is that the structure-function paradigm of proteins is more nuanced than a collection of fixed, static 3D structures. Data from a wide range of measures have revealed varying degrees of conformational flexibility and disorder (e.g., van der Lee et al., 2014), that are not only critical to function, but that can also change during a functional cycle. This picture serves to illustrate the importance to protein metrology of using a range of different measurements, but it also reflects a complex measurement landscape. The increased use of proteins in the Life Sciences, Health, and Food industries complicates the picture further. Which measurements, or combination thereof, are required to demonstrate regulatory compliance (e.g., EU, 2011; EU, 2017) for a given application? One size is unlikely to fit all.

These factors pose important challenges for the metrology community, which seeks to standardise measurement practice, develop reference materials (RMs) and methods, and achieve traceability to the International System of Units (SI). Recent progress has seen new strategies to achieve SI traceability for peptide/protein measurement (e.g., Josephs et al., 2017; Josephs et al., 2019; Cobbaert et al., 2021; Briones et al., 2022), and increased standardisation activities such as the Joint Committee of Traceability in Laboratory Medicine, the International Federation of Clinical Chemistry, and the CCQM (Consultative Committee for Amount of Substance: Metrology in Chemistry and Biology) Working Group on Protein Analysis at the BIPM (International Bureau of Weights and Measures). Much of the activity to date, however, has focussed on traceable measurement of protein quantification and purity. In this Research Topic of 12 papers—6 *Original Research* contributions and 6 *Reviews*/*Perspectives*—we present a snapshot of the current state of the art in protein metrology for structural analysis. Although inevitably not comprehensive, with 67 authors contributing from National Metrology Institutes, industry, and academia from across the globe we hope it is to some degree representative. In many ways, the intention is to be forward-looking; for the Research Topic not only to summarise where we are but where we might need to go to develop a metrology fit for the life sciences.

A common theme of the Research Topic concerns the metrology of protein-based biopharmaceuticals, in particular monoclonal antibody (mAb) therapeutics. Kinumi et al, from the National Metrology Institute of Japan, National Institute of Advanced Industrial Science and Technology (NMIJ/AIST) have developed a new IgG1κ mAb RM, RM6208-a, (AIST-MAB), for the validation and comparison of methods and instrumentation used in the analysis of biopharmaceuticals. They establish the solution concentration of AIST-MAB via amino acid analysis using isotope dilution mass spectrometry (MS) and analyse its physicochemical properties using a range of techniques (e.g., electrophoresis, chromatography methods, peptide/glycan mapping). In Yandrofski et al, the National Institute of Standards and Technology (NIST) in the United States and Agilent Technologies Ltd. review several interlaboratory studies that use another IgG1κ mAb RM, RM8671 (NISTmAb). These studies focus on measurements of both primary and higher-order structure (HOS) of NISTmAb and any post-translational modifications (PMTs) that might have occurred. From the outcomes of each study, they discuss future perspectives, with an emphasis on identifying gaps in the measurement infrastructure used in biopharmaceutical development.

In an original study from NIST, Giddens and Schiel publish a site-specific method to characterise PMTs in NISTmAb. Using a ligand that masks specific sites, they assay oxidation of the unmasked methionine residues in NISTmAb using a range of biophysical approaches (MS, surface plasmon resonance, fluorescence spectroscopy). Their intention is to adapt the method for more general use in the characterisation of PMTs that can change the biological activity of protein-based drugs. Metcalfe from the National Institute for Biological Standards and Control (NIBSC) reviews modern analytical methods for the detection and quantification of free-cysteine residues, which can impact both the stability and efficacy of therapeutic mAbs. He compares spectroscopic (e.g., optical absorption and fluorescence), MS [e.g., isotope labelling and liquid chromatography-MS (LC-MS)], and hybrid methods, again illustrating the range of complementary information provided by using different measures to elucidate a given sample.

mAbs are also used in immunoassays to detect antigens, in clinical diagnostics for example. They are often immobilised by covalent conjugation, but their antigen affinity and specificity can be altered by concomitant changes in HOS. Luckau et al. from the National Measurement Laboratory at LGC and Fleet Bioprocessing Ltd. demonstrate the power of MS methods (LC-MS and hydrogen-deuterium exchange MS) to identify correlations between changes to HOS of mAbs as a result of conjugation and immunoassay performance. Antigen RMs exist to calibrate immunoassays and for cell-based potency testing of mAbs. Because RMs might be stored for long periods before use, they are often lyophilised for stability (the same is true for many biopharmaceuticals). Matejtschuk et al.from NIBSC have investigated the importance of formulation on the preservation of activity of an interleukin-6 RM following the freeze-drying process. The lyophilised material was characterised for several formulations using thermal analysis and scanning electron microscopy, and bioactivity data were compared using a cell-based secreted embryonic alkaline phosphatase (SEAP) assay.

Stability assessment is a central consideration of protein metrology. Properly folded proteins are vital for the safety and efficacy of biopharmaceuticals. A good understanding of protein stability during measurement is also vital to inform experimental design and ensure meaningful and reproducible R&D data. Kwan et al, from the National Physical Laboratory (NPL) in the UK publish a *Perspective* with Douglas Instruments Ltd. summarising methods for the high-throughput screening of conditions that promote stability and reduce aggregation or proteolysis. Instrumentation is now available that enables rapid analysis of low volume samples in multi-well plates to assess properties like hydrodynamic radius (dynamic light scattering), thermal stability (differential scanning fluorimetry), and secondary structure (circular dichroism). Kwok et al. from BLOC Laboratories Ltd., NPL, UCB Pharm Ltd., and the Universities of Bath, Bristol and Waikato report a new thermodynamic model for the quantification of protein conformational flexibility from fluorescence spectroscopy data. Based on the red edge excitation shift (REES) effect of the intrinsic fluorophore, tryptophan, the authors test their new model against several exemplar proteins, including a therapeutic mAb and a *de novo* designed enzyme. This analysis is not only useful for stability screening, but also can help elucidate conformational dynamics that are functional.

Electron paramagnetic resonance (EPR) spectroscopy has proven to be a powerful technique with which to analyse protein conformational dynamics by measuring distance distributions between paramagnetic centres on the nanometre scale. Said et al. from the University of Freiburg evaluate the accuracy of distance measurements made between pairs of spin-correlated radicals in photo-active proteins using out-of-phase electron spin echo envelope modulation (OOP-ESEEM). In doing so, they illustrate the utility of spectral simulation in validating experimentally-derived structural parameters. Continuing with this theme, Russell et al. from the University of St. Andrews provide a comparative review of six different open access simulation/analysis packages with which to extract distance distributions from double electron resonance (DEER) EPR data. In a way that is analogous to making correlated measurements of the same sample using different modalities, the authors reveal how complementary insights can be gained from different methods of analysing the same data.

Proteins and peptides are re-engineered and *de novo* designed for functional properties that are difficult to attain from the synthesis of other organic molecules. Enzymes, for example, achieve challenging chemical transformations with remarkable specificity and selectivity and are therefore desirable targets for use as industrial biocatalysts. Ding et al. from King’s College London review how a combination of structural investigation and computational modelling has aided the rational modification of enzymes. They summarise future perspectives for the field and emphasise the importance of a dynamic picture of protein structure in helping realise the potential of enzyme engineering. *De novo* designed peptides can be active species or can self-assemble into larger structures to serve as biopharmaceuticals or RMs with well-controlled and traceable properties. De Sá Magalhães et al, from University College London, NPL, and Viranova AB, report a method for using an accessible transmission electron microscopy (TEM) approach for determining critical quality attributes of virus-like particles (VLPs). VLPs have been developed as vaccines and drugs, and the authors benchmark their method against VLP model systems.

In summary, this Research Topic illustrates the importance of sound protein metrology to healthcare, industry, measurement science, and fundamental research. It also reflects some of the challenges faced, not only in the context of protein measurement, but also in life sciences metrology more broadly. Looking forward, we acknowledge that HOS is the primary distinction between biological and chemical metrology and must formalise a metrological approach with multimodal, correlated measurement at its heart.
